# Self-Restricted Diet in Pediatric Autism Leading to Vitamin A Deficiency and Severe Photophobia

**DOI:** 10.7759/cureus.54618

**Published:** 2024-02-21

**Authors:** Cory J Dixon, Britton A Ethridge, Aaron P Tillman, Joseph H Sugg

**Affiliations:** 1 Department of Research, Alabama College of Osteopathic Medicine, Dothan, USA; 2 Department of Ophthalmology, Eye Surgical Associates, Dothan, USA

**Keywords:** xerophthalmia, vitamin a deficiency, pediatrics, autism spectrum disorder, photophobia

## Abstract

In developed countries, vitamin A deficiency (VAD) remains rare but is a leading cause of global blindness. We describe the case of a 10-year-old girl with autism spectrum disorder (ASD) initially presenting mild VAD symptoms, escalating to severe photophobia and reluctance to leave a darkened room due to a self-restricted diet of fast-food french fries. A timely examination revealed severe VAD and vitamin A supplementation resolved her symptoms in three weeks. This case highlights the challenge of obtaining accurate patient history in ASD, emphasizing the need for routine dietary discussions and micronutrient testing, especially at ages nine and 13 when the United States Department of Agriculture (USDA) recommends increased vitamin A intake. Early intervention can prevent micronutrient deficiencies in pediatric patients, particularly those with ASD.

## Introduction

Vitamin A is a fat-soluble essential vitamin that plays a critical role in various bodily functions such as vision, immune system support, and cellular differentiation. It is emulsified in the duodenum by pancreatic and intestinal enzymes and then further broken down and stored as retinyl esters in hepatic stellate cell lipid droplets [1,2]. The United States Department of Agriculture (USDA) recommends 300-900 mcg of daily vitamin A intake depending on age and sex [3]. Greater than 99% of the US population maintains adequate vitamin A intake, thanks to retinol, or its derivatives, being widely available from various animal, plant, and dairy sources [4-6]. While vitamin A deficiency (VAD) has been mostly eliminated in developed countries, it remains one of the leading causes of blindness worldwide [7,8]. Severe VAD can lead to ocular symptoms such as conjunctival xerosis, bitot spots, corneal ulceration, xerophthalmic fundus, corneal sclerosis, and night blindness [9]. Worldwide, VAD is typically caused by poor diet or malabsorption, but self-restrictive diets are a more likely cause in developed countries [10-12]. This article presents a case of severe photophobia and behavioral changes occurring due to a self-restricted diet in a child with autism spectrum disorder (ASD).

This article was previously presented as a meeting abstract at the 2023 Alabama College of Osteopathic Medicine-Society of Hospital Medicine (ACOM-SHM) Symposium on November 7, 2023.

## Case presentation

A 10-year-old female with severe autism presented to the clinic with a 10-day history of light sensitivity, bilateral eyelid crusting, epiphora, and redness. Her parents reported that she was being treated with polymyxin/trimethoprim drops which had been recently prescribed by an optometrist. A review of systems was negative for fever, chills, congestion, amaurosis fugax, loss of vision, or extraorbital pain. Initial physical examination revealed erythema of the bilateral ocular adnexa with a quiet and normal lid margin. No conjunctival inflammation was noted. A slit-lamp examination could not be performed due to poor cooperation of the patient (Table 1).

**Table 1:** Initial eye examination of the patient OD: oculus dexter (right eye); OS: oculus sinister (left eye); N/A: not applicable

Eye examination	OD	OS
Ocular adnexa: lid margin	Quiet and normal	Quiet and normal
Ocular adnexa: external	Erythema	Erythema
Conjunctiva	No significant inflammation	No significant inflammation
Slit-lamp examination of the cornea, anterior chamber, irides, and lens	N/A due to poor cooperation	N/A due to poor cooperation

The examination was limited due to poor patient cooperation. The initial plan was for the patient to discontinue polymyxin/trimethoprim and switch to loteprednol/tobramycin 0.3-0.5% drops, one drop three times a day for four to five days and then twice daily for two to three days. Polymyxin/trimethoprim can lead to symptoms such as photosensitivity, eye irritation, and others. Because of this, the patient's parents were educated that polymyxin/trimethoprim drops could have worsened symptoms and were advised to switch to Refresh Liquigel drops and cold compress as needed.

Ten weeks later, the patient presented with severe photophobia, an agitated mood, and a refusal to leave her darkened bedroom. A slit-lamp examination was not possible due to the patient's non-compliance and apparent discomfort. Under anesthesia, the patient's eyes were dilated, and the corneas were stained. The corneal surface was still clear oculus uterque (OU) but demonstrated severely dry epithelium and severe xerosis of the superior conjunctiva. The anterior chamber was quiet and deep with no inflammation. Tension in each eye was 14 mmHg with a Tono-Pen. Optic disc drusen were noted, which was worse oculus dexter (OD), without retinal edema or hemorrhage. No foreign bodies were noted. Refractive error was measured at approximately +4.00 in each eye (Table 2).

**Table 2:** Final eye examination under anesthesia OD: oculus dexter (right eye); OS: oculus sinister (left eye); mmHg: millimeter of mercury

Eye examination	OD	OS
Ocular adnexa: lid margin	Quiet and normal	Quiet and normal
Ocular adnexa: external	Erythema	Erythema
Conjunctiva	Severe xerosis	Severe xerosis
Slit-lamp examination of the cornea	Severely dry epithelium	Severely dry epithelium
Slit-lamp examination of the anterior chamber	Deep and quiet	Deep and quiet
Slit-lamp examination of the irides	Normal iris without rubeosis	Normal iris without rubeosis
Slit-lamp examination of the lenses	Clear lens	Clear lens
Optic disc	Severe drusen	Mild drusen
Eye pressure	14 mmHg	14 mmHg
Refractive error	+4.00	+4.00

Since a complete ocular exam did not reveal a direct cause of her symptoms, the provider initiated an additional discussion with the patient's parents. Further history revealed a self-restricted diet that consisted almost exclusively of fast-food chain french fries.

Ophthalmology recommended immediate vitamin A supplementation and serum confirmation of micronutrient deficiency. A three-week follow-up was completed. During the follow-up, no light sensitivity, bilateral eyelid crusting, epiphora, and redness were seen. Physical examination revealed no erythema of the bilateral ocular adnexa with a quiet and normal lid margin. No conjunctival inflammation was also noted. The patient's parents reported the successful implementation of supplementation via smoothies, and her symptoms had fully resolved. 

## Discussion

Xerophthalmia, as seen here, can have variable degrees of photophobia, conjunctival and corneal xerosis, bitot spots, keratomalacia, nyctalopia, and retinopathy [13]. While VAD is one of the leading causes of preventable blindness in children of developing countries, it is an easily missed clinical diagnosis in more developed countries where it affects less than 1% of the population [6-8]. The USDA recommends 300-900 mcg of daily vitamin A intake depending on age and sex, but the threshold for true deficiency is defined as serum retinol <20 ug/dL [3,6]. No symptomatic threshold has been well described; however, a clear link between VAD and xerophthalmia has been well described in the literature [6-10,13,14]. In the Journal of Nutrition, Sommer uses "ancient" accounts of the signs and symptoms of xerophthalmia to support the idea that advanced xerophthalmia is pathognomonic for VAD [14].

Food selectivity and restricted diet comprise a wide range of behaviors including restricted calorie intake, unpredictable food refusal/preference, food-related rituals or obsessions, and behavioral problems at mealtimes [3]. Transient food selectivity is common in pediatric patients but is more likely to be consistent or extreme in pediatric autistic patients [15]. According to Page et al., food restriction is estimated to be up to 89% more likely in patients with ASD compared to pediatric patients not on the autism spectrum [16]. A 2021 systematic review examined the relationship between avoidant restrictive food intake disorder (ARFID) and ASD, or the broad autism phenotype [17]. Examples of ARFID include vitamin C deficiency and VAD [17]. A total of 76 cases were reviewed, and an incidental finding was that VAD was the second most common deficiency and every VAD case was found secondary to ophthalmic complaints at a rate of 17.1% suggesting that the true rate is higher [17]. This case demonstrates how difficulty eliciting patient history and performing an adequate exam can significantly affect health outcomes and delay appropriate care, especially in patients with ASD. While this patient's symptoms were reversible, some cases are not found until significant irreversible corneal damage has already occurred [9,11,14].

## Conclusions

Due to the minor prevalence of symptomatic micronutrient deficiency in the general population, an unknown number of preventable ocular diseases still occur in the United States, particularly in patients with ASD. This case highlights the importance of a thorough review of dietary intake in all patients with atraumatic visual disturbances, especially those with a tendency to self-restrict their diets. Previous evidence-based revisions to the clinical workflow for pediatric ASD patients have focused on intervening based on identifying and then addressing identifiable "antecedents of the feeding issues" but have largely not addressed preventative screening for micronutrient deficiencies. The authors conclude that routine discussions on dietary intake followed by micronutrient testing could occur for all pediatric patients, especially those with ASD. As outlined by the USDA, two important milestones this new intervention could be added to are the two ages that the recommended daily intake for vitamin A increases, nine and 13 years old.
